# Active Disturbance Rejection Control for an automotive suspension system based on parameter tuning using a fuzzy technique

**DOI:** 10.1371/journal.pone.0313104

**Published:** 2025-01-15

**Authors:** Tuan Anh Nguyen

**Affiliations:** Faculty of Mechanical Engineering, Thuyloi University, Hanoi, Vietnam; Federal University of Technology - Parana, BRAZIL

## Abstract

Road surface roughness is the cause of vehicle vibration, which is considered a system disturbance. Previous studies on suspension system control often ignore the influence of disturbances while designing the controller, leading to system performance degradation under severe vibration conditions. In this work, we propose a control method to improve active suspension performance that reduces vehicle vibration by eliminating the influence of road disturbances. The proposed method is formed based on the combination of an Active Disturbance Rejection Control (ADRC) technique with control coefficients tuned by a dynamic fuzzy technique formed based on special membership functions called Active Disturbance Rejection Control Based on Fuzzy (ADRCBF). An Extended State Observer (ESO) estimates state variables and disturbances. The performance of the proposed controller is evaluated through the numerical simulation process with three different cases. According to the calculation results, the acceleration and displacement of the sprung mass are significantly reduced when the suspension system is controlled by the proposed technique, compared with the passive suspension system and the active suspension system controlled by a Proportional-Integral-Derivative (PID) technique. In addition, the suspension travel follows the road disturbance with a small error. The error estimated by the ESO does not exceed 3.5% (for sinusoidal and random excitation). In general, system adaptation is ensured under many investigated conditions based on tuning the controller parameters by the soft computing method.

## 1. Introduction

Automotive suspension systems have been researched and developed for many years. While traditional suspension systems, such as mechanical or passive suspension, only consist of coil springs and hydraulic dampers with constant stiffness, advanced suspension systems offer high efficiency through varying damper and spring stiffness. According to Yu et al., active suspension systems have improved passenger stability, comfort, and travel safety [1]. Today, automotive suspension systems are divided into three types: passive suspension, semi-active suspension, and active suspension. According to Ferhath and Kasi, active suspension systems have provided superior performance compared to remaining systems [2]. However, the structure of active suspension systems is more complicated, leading to increased weight and cost. The active suspension system is equipped with a separate actuator (hydraulic or electronic) located next to the coil spring and hydraulic damper. This actuator is independently controlled by a controller based on sensor signals [3]. The actuator control reduces vehicle vibrations, i.e., body displacements and accelerations [4].

### 1.1. Literature review

Many control methods have been applied to control the performance of active suspension systems. The scope of application of the methods depends on the object and the control purpose. We can apply the PID technique if the system is considered linear with single input and single output (SISO). In [5], Dangor et al. designed an evolutionary algorithm to tune the parameters for the PID controller of the suspension system. This is a general name for optimization techniques, including Particle Swarm Optimization (PSO), Genetic Algorithm (GA), and Differential Evolution (DE). In [6], Nagarkar et al. used the GA to find the ideal parameters for the controller based on multi-objective optimization, which consists of sprung mass acceleration, suspension travel, tire deflection, and control force. The GA mechanism shaped based on six steps was shown in [7] by Nguyen et al. They used the Root Mean Square (RMS) value of the sprung mass displacement as an objective function to find the ideal parameters so that the objective function was minimized. The number of unknowns increases when replacing the conventional PID technique with Fractional Order PID (FOPID). Gad et al. designed the GA to identify five suspension controller parameters. The simulation results in [8] showed that displacement and acceleration were reduced when applying the GA-FOPID technique instead of the conventional PID. The genetic algorithm is only effective in a local search range, whereas the PSO’s range is more extensive [9]. Abdulzahra and Abdalla introduced an optimization calculation technique based on the PSO mechanism called Artificial Bee Colony (ABC) [10]. The objective of the work done in [10] was to find the appropriate parameters for the PID controller, which was applied to control the active suspension system. An extension of the work was shown in [11], where Zahra and Abdalla applied the above technique to tune the parameters of the FOPID controller. Based on the behavior of ants, Mughees and Mohsin designed a technique called Ant Colony Optimization (ACO) to find the ideal control parameters [12]. An application in vibration control called the Advanced Firefly Algorithm (AFA) was proposed in [13] by Talib et al. Simulation results showed that the sprung mass acceleration was reduced by more than 50% when the above technique was combined with PID control. Karam and Awad presented another application, the Whales Optimization Algorithm (WOA), in [14], where they optimized the parameters for a quarter suspension system model. The optimal parameters were only valid in one or some specific cases, i.e., the system performance may degrade when applied to other cases. To solve this problem, Nguyen proposed using a fuzzy technique to tune the parameters of the PI controller [15]. Han et al. [16] designed an adaptive fuzzy controller based on changes in road surface conditions to increase the system’s adaptability. The fuzzy-PID controller proposed in [17] was formed based on three layers with dynamic inputs. Simulation results showed that the vehicle body displacement was significantly reduced when applying this technique compared to the passive suspension system.

The PID control structure is quite simple, and this controller is highly systematic. However, it is only suitable for SISO systems. This algorithm cannot be applied to systems formed based on multiple inputs and multiple outputs (MIMO). A Linear Quadratic Regulator (LQR) technique was proposed in [18] to solve the above problem. The goal of the LQR algorithm was to minimize the cost function, according to Rodriguez-Guevara et al. [19]. In [20], Manna et al. applied the ACO technique to tune the weight matrices of the system. An application of the GA to finding the ideal coefficients of LQR control was performed by Pereira et al. [21]. The ACO tuning method offered many advantages over classical tuning. A comparison between the LQR and H_∞_ algorithms was done in [22] by Kaleemullah et al. They concluded that the performance of the LQR technique was slightly higher than that of H_∞_. However, this is only true under certain conditions. In addition, the LQR technique requires multiple measurements of state variables, which are affected by sensor noise.

Compared with conventional control techniques, robust control methods effectively control nonlinear systems affected by disturbances. In [23], Nguyen designed an essential Sliding Mode Control (SMC) algorithm to control a quarter suspension system that accounted for the influence of hydraulic actuators. A method for calculating the optimal parameters for SMC was presented in [24]. In [25], Chen et al. presented an improved optimal sliding mode controller based on an extended controller formulation. Ovalle et al. presented an application of chattering reduction in [26], although the influence was still significant. In [27], Ghadiri and Montazeri designed an adaptive integral terminal SMC to control a nonlinear suspension system affected by external disturbances. The stability of the proposed system in [27] was evaluated using the Lyapunov theory. Instead of applying conventional linear sliding surfaces [28], Wijaya et al. proposed a natural logarithm sliding surface to improve the performance of the SMC mechanism [29]. An adaptive disturbance observer method based on a practical terminal SMC framework was presented by Wang et al. in [30]. Previously, a disturbance observer formed based on system uncertainty and error was introduced in [31] by Deshpande et al. Although the SMC technique provides stability for the system, the chattering phenomenon still exists and causes some adverse effects. To solve this problem, Taghavifar et al. proposed an improved sliding surface, which was considered a core component of the fuzzy neural network based on the sliding mode control technique [32]. Another solution was presented in [33], which described an integrated controller formed based on the combination of SMC and PID with the parameters optimized by an in-loop algorithm. Combining the SMC technique with the fuzzy computing method effectively reduced system error, although it could not completely eliminate the chattering phenomenon [34, 35]. An integrated control technique between LQR and SMC was presented in [36] by Bai and Wang. Compared with the conventional LQR, this integrated algorithm provided superior performance, as verified by the CARSIM simulation. A high-performance combined mechanism was presented by Nkomo et al. in [37]. They designed a controller integrated with sliding mode control and backstepping control techniques to improve performance. Experimental results on a simple model showed that the performance of the proposed algorithm was superior to that of the component techniques. Overall, the structure of the SMC mechanism is quite complex, and the chattering phenomenon still exists even when the algorithm has been improved by incorporating soft computing techniques.

Practical systems are exposed to the influence of external disturbances. The measurement of disturbance signals is costly and causes significant system errors. In [38], Li et al. introduced the Active Disturbance Rejection Control (ADRC) technique to control a nonlinear suspension system to eliminate the influence of external disturbances. According to Fareh et al., ADRC could overcome the disadvantages of PID control under a significant change in load conditions [39]. The basic structure of an active disturbance rejection controller consists of a Tracking Differentiator (TD), an Extended State Observer (ESO), and a feedback control law. A combination of ADRC and SMC was introduced in [40] by Suhail et al. Simulation results showed that the system error was significantly reduced when applying the robust control method compared to the conventional PID. An application of ADRC to control a suspension system model with seven degrees of freedom was introduced by Muhammed et al. [41]. However, the obtained results showed a slight chatter in the output signal. An improved ADRC mechanism, which was formed by the combination of the SMC technique, fuzzy computing, and linear ESO, was proposed in [42] by Wang et al. Although the ADRC algorithm can perform highly in rejecting external disturbances, the system error may increase if the coefficients are not selected appropriately. The simulation results in [43] showed that the control performance of the ADRC algorithm was degraded compared to intelligent PD control.

Some control methods that provide high efficiency to the system based on data-driven control mechanisms should be referred to in [44–46]. Some applications involving hysteresis quantified control [47], finite-time SMC [48], anti-disturbance state estimation [49], and event-triggered H_∞_ [50] can be applied to improve the performance of active suspension systems. Although the efficiency of the algorithms is high, their structure is quite complex.

### 1.2. Motivation and new contribution

The control techniques mentioned in the previous subsection have certain advantages for controlling automobile suspension systems. However, some drawbacks still exist, which are considered research gaps. Firstly, traditional PID control fails to be applied to MIMO systems. Although the structure of PID control is simple, its performance is insufficient to improve system quality in many cases. Secondly, conventional LQR control requires all output signals to be obtained by physical sensor measurement, which is costly and increases the error (due to sensor noise). Thirdly, the SMC mechanism causes chattering, degrading the system’s quality. Finally, some applications of classical ADRC do not provide sufficient performance to control the system, causing significant errors in some harsh working conditions. This is because the choice of fixed control parameters reduces the system’s adaptability. Finding solutions to address the above problems motivates the work presented in this article. Comparisons between the control methods are given in Table 1.

**Table 1:** Comparison of control methods.

Criterion	Proposed method	PID	LQR	Classical ADRC	SMC
Response speed	High	High with little overshoot	Medium	Medium	High
Adaptability	High	Medium	Medium	Medium	High
Phase difference	Inconsiderable	Medium	Medium	Medium	Inconsiderable
Chattering	Inconsiderable	Medium	Medium	Inconsiderable	Significant
Tracking errors	Inconsiderable	High	High	Medium	Low
Affected by disturbances	Slight	Significant	Significant	Slight	Slight
Algorithm structure	Simple	Simple	Simple	Simple	Complicated
Scope of application	Linear and nonlinear systems with MIMO	Linear system with SISO	Linear system with MIMO	Linear and nonlinear systems with MIMO	Linear and nonlinear systems with MIMO

In this article, we propose designing a hybrid control technique that combines the ADRC algorithm with an advanced fuzzy computing method to solve the existing problems. Compared to conventional control methods, ADRC offers high efficiency in controlling systems affected by external disturbances. In addition, the influence of sensor noise is eliminated based on estimating values instead of measuring them by sensors. The algorithm’s structure is also simple, making the design process more straightforward. The article’s novelty lies in its application of a dynamic fuzzy computing method to improve system response, while the structure of the ADRC algorithm is almost unaffected. The proposed hybrid algorithm can be applied to SISO and MIMO systems (1^st^ problem). The system output signals are estimated by the ESO instead of being directly measured by sensors, while an augmented state variable observes the system disturbance. This helps to reduce system errors and operating costs (2^nd^ problem). Compared with complex sliding mode controllers, the ADRC structure is more straightforward and does not cause chattering (3^rd^ problem). To increase the adaptability of the system to the dynamic changes of road disturbances, the control parameters need to be tuned using dynamic fuzzy techniques instead of fixed values, which is considered a significant new contribution to solving the final problem.

The structure of the article is organized into four sections. Literature reviews and new contributions are mentioned in the Introduction section. The second section presents the system’s mathematical method and the control algorithm. The computational results are discussed and evaluated in the third section. Finally, some comments are given in the Conclusion section.

## 2. Mathematical method

This section presents the mathematical model of the dynamic system and the control algorithm.

### 2.1. Dynamic model

**Fig 1:**
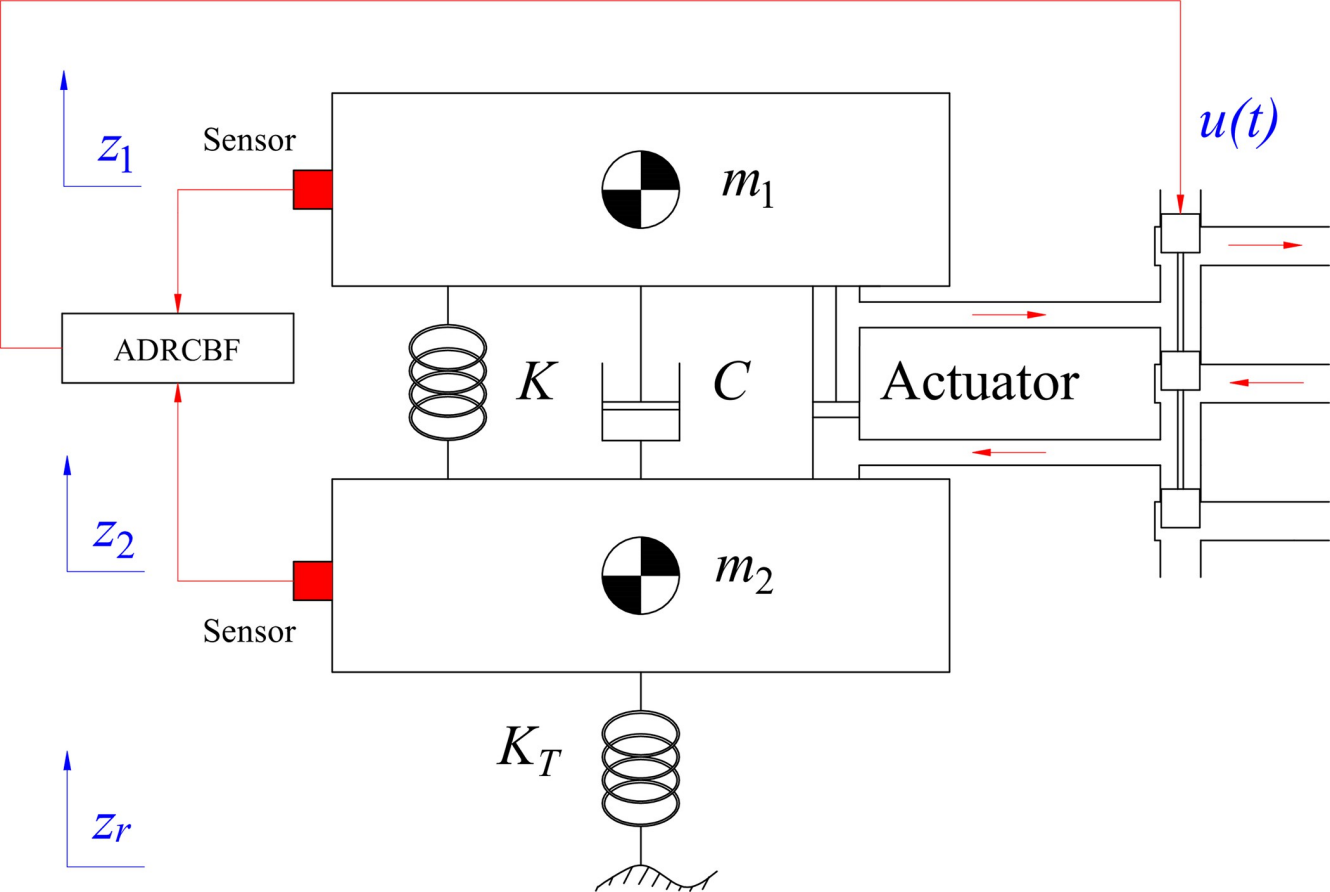
Quarter dynamic model.


In the present study, we introduce the utilization of a quarter-dynamic model, as depicted in Fig 1. This model is designed to simplify the computational process. Regarding half- or full-dynamic models, each actuator can be controlled by a separate electronic control module established from the same algorithm or different algorithms. The vehicle vibration is shown by Eqs (1) and (2) using D’Alembert’s principle.

$$F_{im1}=F_{K}+F_{C}+F_{A}$$ (1)

$$F_{im2}=F_{KT}-F_{K}-F_{C}-F_{A}$$ (2)

Where *F*_*im*1_ is an inertia force of the sprung mass, *F*_*im*2_ is an inertia force of an unsprung mass, *F*_*K*_ is a suspension spring force, *F*_*C*_ is a suspension damper force, *F*_*KT*_ is a tire spring force, and *F*_*A*_ is a control force of an actuator. These forces are calculated according to the linked Eqs from (3) to (7).


$$F_{im1}=m_{1}{\ddot{z}}_{1}$$ (3)

$$F_{im2}=m_{2}{\ddot{z}}_{2}$$ (4)

$$F_{K}=K(z_{2}-z_{1})$$ (5)

$$F_{C}=C({\dot{z}}_{2}-{\dot{z}}_{1})$$ (6)

$$F_{KT}=K_{T}(z_{r}-z_{2})$$ (7)

In the initial state, the sprung and unsprung mass displacement is zero. The effect of the load is determined at the initial position of the spring and damper. The symbols mentioned in the above equations are listed in Table 2.

**Table 2:** Vehicle specifications.

Symbol	Meaning	Unit	Value
*m*_1_	Sprung mass	kg	380
*m*_2_	Unsprung mass	kg	40
*K*	Spring stiffness	N/m	38500
*C*	Damper coefficient	Ns/m	3050
*K*_*T*_	Tire stiffness	N/m	170000
*z*_1_	Sprung mass displacement	m	-
*z*_2_	Unsprung mass displacement	m	-
*z*_*r*_	Road disturbance	m	-

Substituting Eqs (3) to (7) into (1) and (2), we get (8) and (9).


$$m_{1}{\ddot{z}}_{1}=-Kz_{1}-C{\dot{z}}_{1}+Kz_{2}+C{\dot{z}}_{2}+F_{A}$$ (8)

$$m_{2}{\ddot{z}}_{2}=Kz_{1}+C{\dot{z}}_{1}-(K+K_{T})z_{2}-C{\dot{z}}_{2}-F_{A}+K_{T}z_{r}$$ (9)

Let the state variables be according to (10). Taking the derivative of the state variables, we get (11÷14).


$${\left[\begin{array}{cc} \begin{array}{cc} \begin{array}{cc} x_{1} & x_{2} \end{array} & x_{3} \end{array} & x_{4} \end{array}\right]}^{T}={\left[\begin{array}{cc} \begin{array}{cc} \begin{array}{cc} z_{1} & {\dot{z}}_{1} \end{array} & z_{2} \end{array} & {\dot{z}}_{2} \end{array}\right]}^{T}$$ (10)

$${\dot{x}}_{1}=x_{2}$$ (11)

$${\dot{x}}_{2}=-\frac{K}{m_{1}}x_{1}-\frac{C}{m_{1}}x_{2}+\frac{K}{m_{1}}x_{3}+\frac{C}{m_{1}}x_{4}+\frac{1}{m_{1}}F_{A}$$ (12)

$${\dot{x}}_{3}=x_{4}$$ (13)

$${\dot{x}}_{4}=\frac{K}{m_{2}}x_{1}+\frac{C}{m_{2}}x_{2}-\frac{K+K_{T}}{m_{2}}x_{3}-\frac{C}{m_{2}}x_{4}-\frac{1}{m_{2}}F_{A}+\frac{K_{T}}{m_{2}}z_{r}$$ (14)

The model of the passive suspension system is rewritten according to (15) with *F*_*A*_ = 0.


$$[\begin{array}{c} {\dot{x}}_{1}\\ {\dot{x}}_{2}\\ {\dot{x}}_{3}\\ {\dot{x}}_{4} \end{array}]=[\begin{array}{cccc} 0 & 1 & 0 & 0\\ -\frac{K}{m_{1}} & -\frac{C}{m_{1}} & \frac{K}{m_{1}} & \frac{C}{m_{1}}\\ 0 & 0 & 0 & 1\\ \frac{K}{m_{2}} & \frac{C}{m_{2}} & -\frac{K+K_{T}}{m_{2}} & -\frac{C}{m_{2}} \end{array}][\begin{array}{c} x_{1}\\ x_{2}\\ x_{3}\\ x_{4} \end{array}]+[\begin{array}{c} 0\\ \frac{1}{m_{1}}\\ 0\\ -\frac{1}{m_{2}} \end{array}]F_{A}+[\begin{array}{c} 0\\ 0\\ 0\\ \frac{K_{T}}{m_{2}} \end{array}]z_{r}$$ (15)

### 2.2 Control algorithm

**Fig 2:**
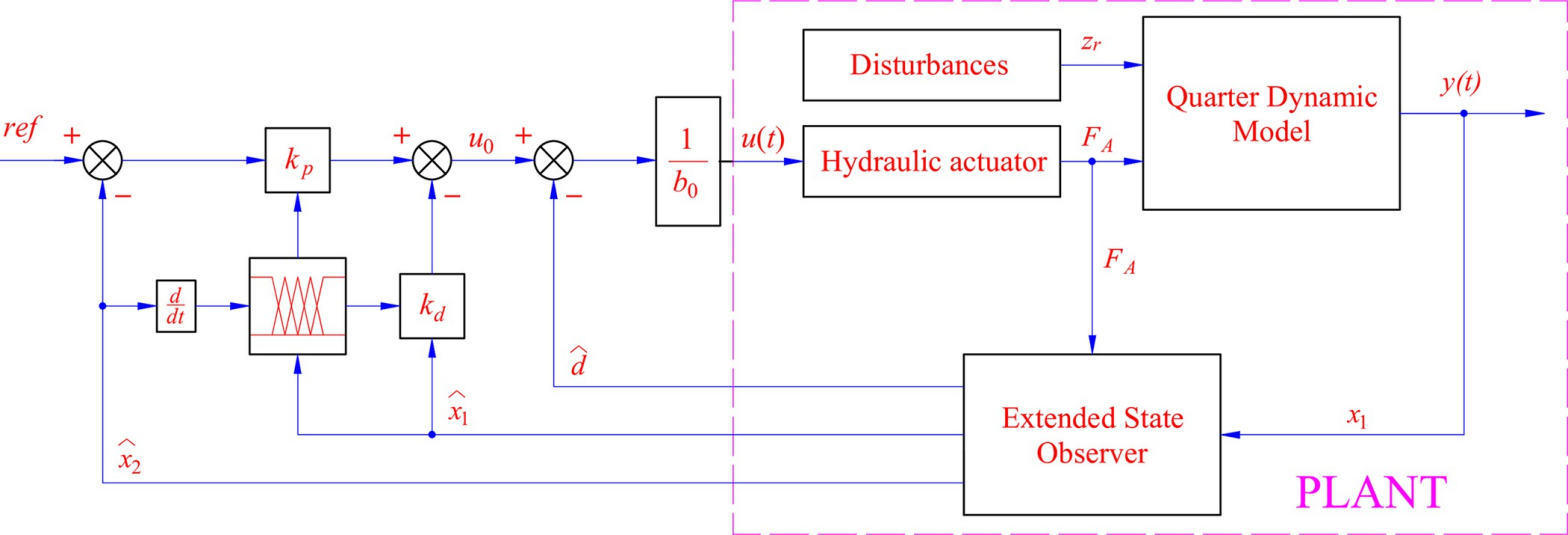
Control scheme.

A control algorithm called ADRC is proposed in this work to control the performance of the active suspension system. The algorithm is formed by combining a proportional stage, a derivative stage, and an ESO. The ESO estimates the changes in the output state variables instead of measuring them directly with sensors. In addition, the road disturbance is also determined by observing the signal, which is performed by the ESO (Fig 2).

Let *e* (16) be the error between the observed and actual signals. The road disturbance is determined through an augmented variable, according to (17). The system (15) is rewritten as (18). The coefficient matrices are explained according to (19), where *β*_*i*_ are the gain coefficients.


$$e={\hat{x}}_{1}-x_{1}$$ (16)

$${\hat{x}}_{5}=z_{r}$$ (17)

$$[{\dot{\hat{x}}}_{i}]=\hat{A}[{\hat{x}}_{i}]+\hat{B}F_{A}+\hat{\beta }e$$ (18)

$$\begin{array}{l} \hat{A}=\left[\begin{array}{ccccc} 0 & 1 & 0 & 0 & 0\\ -\frac{K}{m_{1}} & -\frac{C}{m_{1}} & \frac{K}{m_{1}} & \frac{C}{m_{1}} & 0\\ 0 & 0 & 0 & 1 & 0\\ \frac{K}{m_{2}} & \frac{C}{m_{2}} & -\frac{K+K_{T}}{m_{2}} & -\frac{C}{m_{2}} & \frac{K_{T}}{m_{2}}\\ 0 & 0 & 0 & 0 & 0 \end{array}\right]\\ \hat{B}={\left[\begin{array}{ccccc} 0 & \frac{1}{m_{1}} & 0 & -\frac{1}{m_{2}} & 0 \end{array}\right]}^{T}\\ \hat{\beta }={\left[\begin{array}{ccccc} -\beta_{1} & -\beta_{2} & -\beta_{3} & -\beta_{4} & -\beta_{5} \end{array}\right]}^{T} \end{array}$$ (19)

The control force of the hydraulic actuator (*F*_*A*_) is denoted as *x*_6_. The dynamics of the actuator are fully described in [23]. In this work, we use a linearized model of the actuator (20) to simplify the problem [35].


$${\dot{x}}_{6}\approx \gamma_{1}u(t)-\gamma_{2}x_{6}-\gamma_{3}(x_{2}-x_{4})\overset{x_{2}\approx {\hat{x}}_{2}}{\underset{x_{4}\approx {\hat{x}}_{4}}{\to }}\gamma_{1}u(t)-\gamma_{2}x_{6}-\gamma_{3}({\hat{x}}_{2}-{\hat{x}}_{4})$$ (20)

In this work, the object to be controlled is the sprung mass displacement. Combining (8) with (18) and (20), we get (21). The control signal *u*(*t*) is determined according to (22) and (23), where b0 is the critical gain, *r* is the reference signal, and d is the total disturbance.


$${\ddot{z}}_{1}=-\frac{K}{m_{1}}{\hat{x}}_{1}-\frac{C}{m_{1}}{\hat{x}}_{2}+\frac{K}{m_{1}}{\hat{x}}_{3}+\frac{C}{m_{1}}{\hat{x}}_{4}+\frac{1}{m_{1}}{\hat{x}}_{6}=\hat{d}+b_{0}{\hat{x}}_{6}$$ (21)

$$u(t)=\frac{u_{0}-\hat{d}}{b_{0}}$$ (22)

$$u_{0}=k_{p}(r-{\hat{x}}_{1})-k_{d}{\hat{x}}_{2}$$ (23)

In this work, the coefficients *k*_*p*_ and *k*_*d*_ are tuned by a dynamic fuzzy technique instead of finding a specific value. The goal is to improve the system’s adaptability to changes in state variables. The structure of the fuzzy controller, consisting of multiple inputs and one output, is shown as follows:

***C***_**1**_: IF *x*_1_ = *A*_11_ and *x*_2_ = *A*_12_ and *x*_3_ = *A*_13_ and … and *x*_*m*_ = *A*_1*m*_, THEN *y* = *B*_1_ or

***C***_**2**_: IF *x*_1_ = *A*_21_ and *x*_2_ = *A*_22_ and *x*_3_ = *A*_23_ and … and *x*_*m*_ = *A*_2*m*_, THEN *y* = *B*_2_ or

***C***_**3**_: IF *x*_1_ = *A*_31_ and *x*_2_ = *A*_32_ and *x*_3_ = *A*_33_ and … and *x*_*m*_ = *A*_3*m*_, THEN *y* = *B*_3_ or

…

***C***_***n***_: IF *x*_1_ = *A*_*n*1_ and *x*_2_ = *A*_*n*2_ and *x*_3_ = *A*_n3_ and … and *x*_*m*_ = *A*_*nm*_, THEN *y* = *B*_*n*_

Let *μ*_*Aki*_(*x*) and *μ*_*Bk*_(*y*) be the membership functions of *A*_*ki*_ and *B*_*k*_, respectively. The satisfaction of the composition rule is determined by (24), where *x*_0_ is the explicit value of the input linguistic variable. The membership function of the output value *μ*_*B’*_(*y*) is determined by the Prod method, which is shown in (25). The defuzzification process is performed using the weighted average method.


$$H_{k}=\min(\mu_{Aki}(x_{0}))$$ (24)

$$\mu_{B'k}(y)=\mu_{Ak}(x_{0})\mu_{Bk}(y)=H_{k}\mu_{Bk}(y)$$ (25)

**Fig 3:**
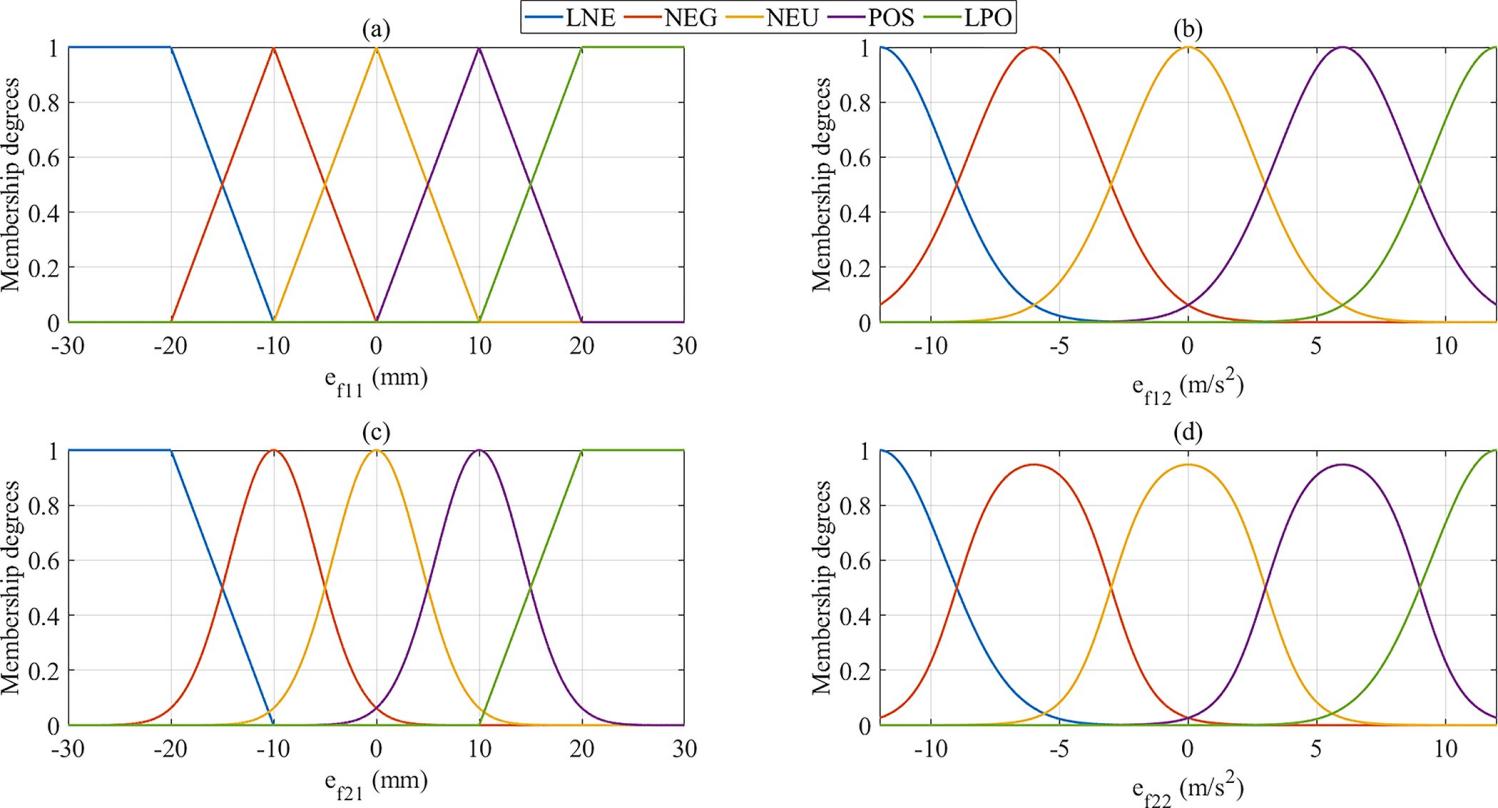
Membership functions. (a) The first input (*k*_*p*_ coefficient). (b) The second input (*k*_*p*_ coefficient). (c) The first input (*k*_*d*_ coefficient). (d) The second input (*k*_*d*_ coefficient).

The fuzzy controllers’ inputs are the changes in sprung mass displacement (1^st^ input) and sprung mass acceleration (2^nd^ input). The structure of the first fuzzy controller, which is used to tune the proportional coefficient, is shown in Fig 3A and 3B. Regarding the first input (Fig 3A), Triangular Membership Functions (TRIMFs) are used to determine the changes in sprung mass displacement in a small range. If the value of the first state variable (*x*_1_) is significant, the membership degree will reach saturation using Trapezoidal Membership Functions (TRAPMFs). The slope of the triangular and trapezoidal functions is significant, increasing the system’s response speed. The changes in acceleration (2^nd^ input) are sensitive, leading to significant changes in smoothness and comfort. Therefore, triangular and trapezoidal membership functions should be replaced by Gaussian Membership Functions (GAUSSMFs).

The number of membership functions of the second fuzzy algorithm, which is used to tune the derivative coefficient, is similar to that of the first fuzzy algorithm. However, the structure of the membership functions is different. Triangular membership functions should be replaced by Gaussian membership functions to reduce the overshoot phenomenon (Fig 3C). The membership degree will reach saturation once the sprung mass displacement is large. A significant difference is seen in Fig 3D when replacing Gaussian functions with Product of Two Sigmoidal Membership Functions (PSIGMFs). Compared with the above membership functions, the slope of PSIGMFs is slightly higher, which helps to reduce the influence of the overshoot phenomenon. The abbreviations are explained: LNE is largely negative, NEG is negative, NEU is neutral, POS is positive, and LPO is largely positive. The mathematical models of TRIMFs, TRAPMFs, GAUSSMFs, and PSIGMFs are shown in (26), (27), (28), and (29), respectively. A recent publication on fuzzy control for mechatronics systems was presented in [51], specifically the Takagi–Sugeno model and stability analysis when designing controllers.


$$\mu (x;a,b,c)=\max(\min(\frac{x-a}{b-a},\frac{c-x}{c-b}),0)$$ (26)

$$\mu (x;a,b,c,d)=\max(\min(\frac{x-a}{b-a},1,\frac{d-x}{d-c}),0)$$ (27)

$$\mu (x;\sigma ,c)=e^{-\frac{{\left(x-c\right)}^{2}}{2\sigma^{2}}}$$ (28)

$$\mu (x;a,c)=\frac{1}{1+e^{-a(x-c)}}$$ (29)

**Fig 4:**
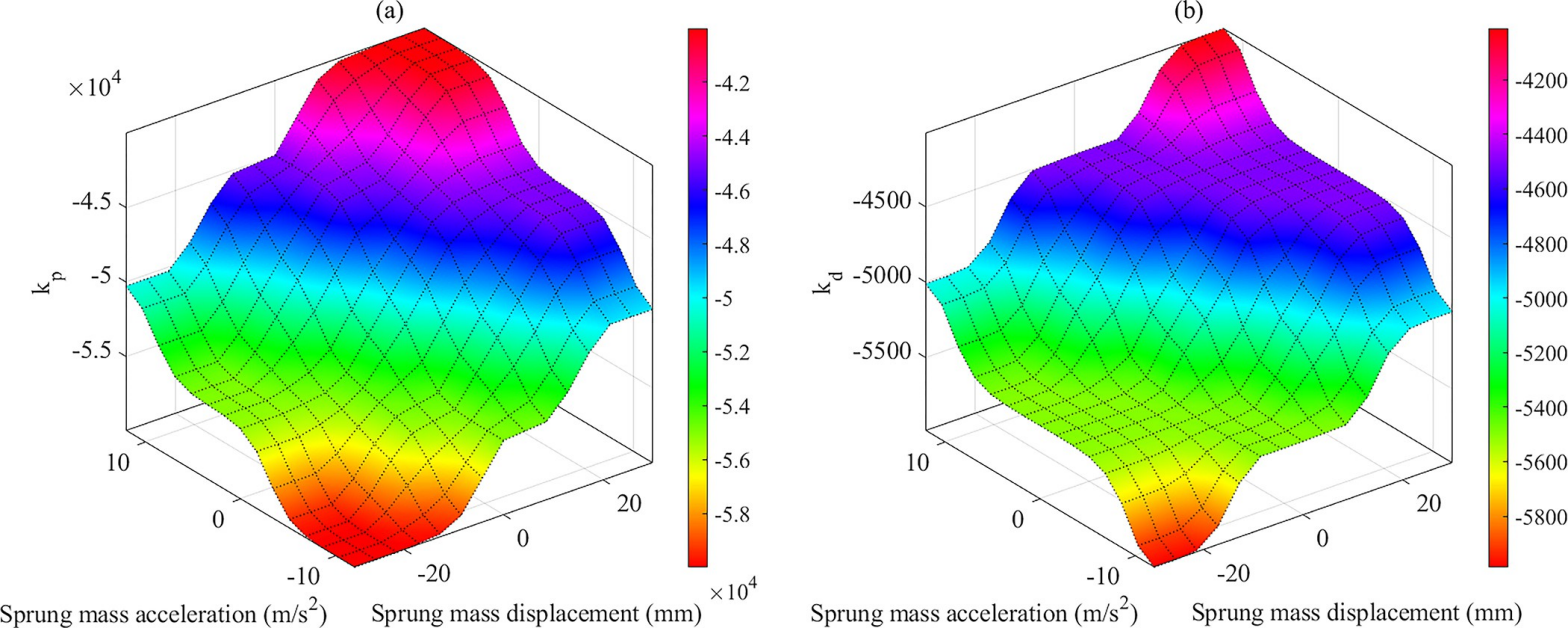
Fuzzy surfaces. (a) 1^st^ fuzzy controller (*k*_*p*_). (b) 2^nd^ fuzzy controller (*k*_*d*_).

The relationship between the input and output of the fuzzy algorithm is illustrated by the 3D graphs in Fig 4. These fuzzy surfaces are formed based on the fuzzy rules, which are listed in Tables 3 and 4. The parameters of the fuzzy controller are selected based on the experience gained from previous simulations. The experimental process to find the parameters was repeated many times based on the cases mentioned in the third section. The selected parameters are shown in the subplots of Fig 3. The fuzzy rules are selected to satisfy the following requirements: Firstly, reduce the sprung mass displacement and acceleration quickly but without causing any adverse effects on the vehicle vibration. Secondly, the overshoot influence should be removed to smooth the vibration. Finally, when the vibration is significant, determine the saturation state. Several optimization search algorithms, such as the Advanced Firefly Algorithm [52], the Cuckoo Search Algorithm [53], and others, can be applied to determine the ideal parameters for the fuzzy controller.

**Table 3:** Fuzzy rules (*k*_*p*_).

1^st^ input	2^nd^ input	Output	1^st^ input	2^nd^ input	Output	1^st^ input	2^nd^ input	Output
LNE	LNE	LNE	NEG	LPO	POS	POS	POS	POS
LNE	NEG	LNE	NEU	LNE	NEG	POS	LPO	LPO
LNE	NEU	NEG	NEU	NEG	NEG	LPO	LNE	NEU
LNE	POS	NEG	NEU	NEU	NEU	LPO	NEG	POS
LNE	LPO	NEU	NEU	POS	POS	LPO	NEU	POS
NEG	LNE	LNE	NEU	LPO	POS	LPO	POS	LPO
NEG	NEG	NEG	POS	LNE	NEG	LPO	LPO	LPO
NEG	NEU	NEG	POS	NEG	NEU			
NEG	POS	NEU	POS	NEU	POS			

**Table 4:** Fuzzy rules (*k*_*d*_).

1^st^ input	2^nd^ input	Output	1^st^ input	2^nd^ input	Output	1^st^ input	2^nd^ input	Output
LNE	LNE	LNE	NEG	LPO	POS	POS	POS	POS
LNE	NEG	NEG	NEU	LNE	NEG	POS	LPO	POS
LNE	NEU	NEG	NEU	NEG	NEG	LPO	LNE	NEU
LNE	POS	NEG	NEU	NEU	NEU	LPO	NEG	POS
LNE	LPO	NEU	NEU	POS	POS	LPO	NEU	POS
NEG	LNE	NEG	NEU	LPO	POS	LPO	POS	POS
NEG	NEG	NEG	POS	LNE	NEG	LPO	LPO	LPO
NEG	NEU	NEG	POS	NEG	NEU			
NEG	POS	NEU	POS	NEU	POS			

This work uses "data-driven" to serve the analysis and evaluation process. In addition, the system output data is used as input for the fuzzy controller to calculate the parameters for PD control. Regarding data-driven control, the system specifications are set as constraints. The evaluation of system stability should be referred to in [54].

## 3. Result and discussion

Road excitation (disturbance) is considered the input of the simulation problem. Three types of excitations are used to investigate the vehicle vibration, including sinusoidal, step, and random excitations. The control target is to reduce vehicle vibration, that is, to reduce vehicle body acceleration and displacement. Although sprung mass acceleration and displacement are closely related, examining both factors is necessary to evaluate vehicle comfort fully. In addition, the active suspension system needs to work harder to eliminate vibration, which results in increased suspension travel. Therefore, the system performance is evaluated based on changes in all three above factors, including sprung mass displacement, sprung mass acceleration, and suspension travel. The results obtained by the proposed algorithm (ADRCBF) are compared with those of ESO, PID, and Passive.

### 3.1. Sinusoidal excitation

**Fig 5:**
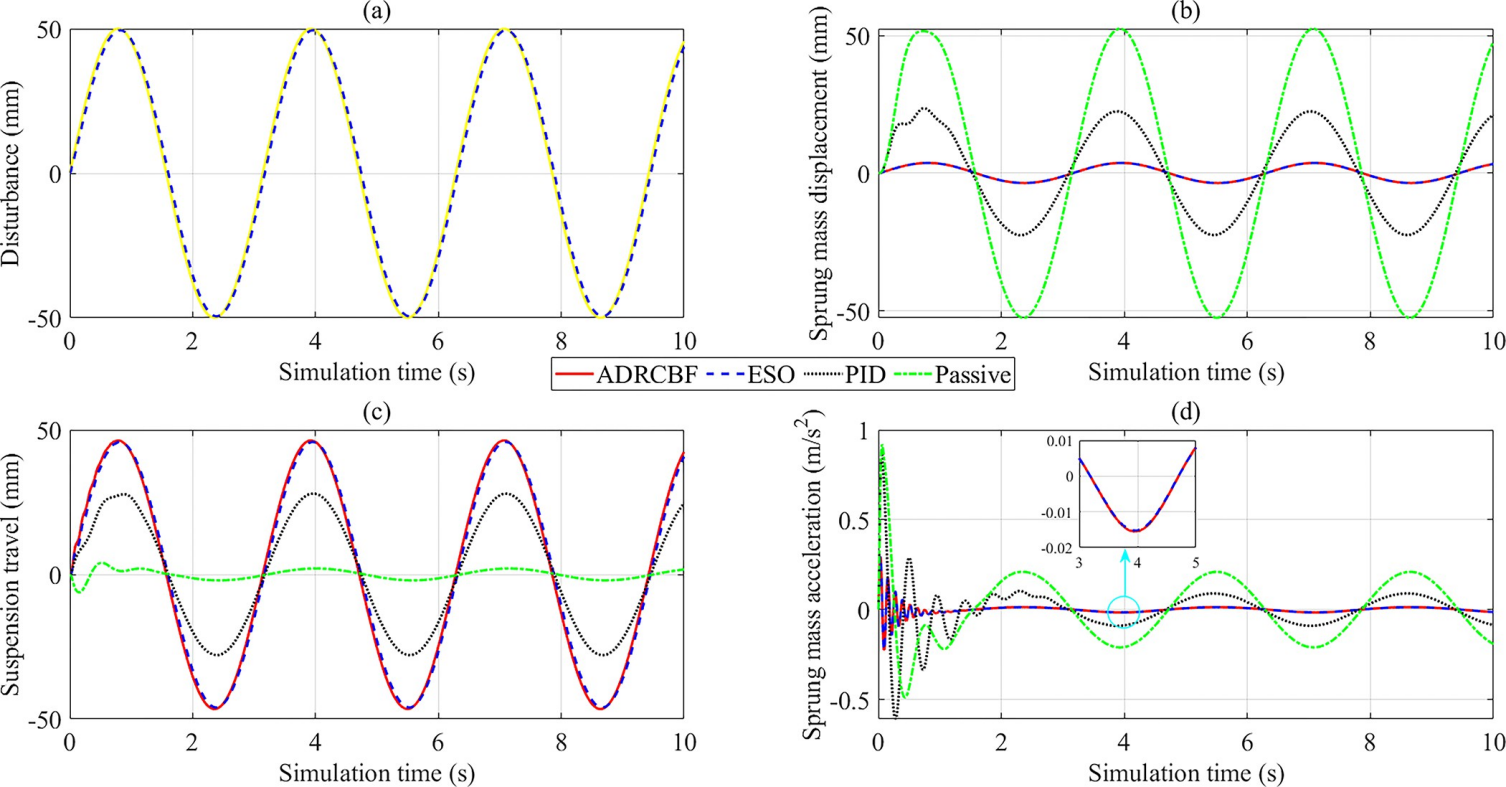
Simulation results (sinusoidal excitation). (a) Observed disturbance. (b) Sprung mass displacement.(c) Suspension travel. (d) Sprung mass acceleration.

The change in output state variables is illustrated by subplots in Fig 5. The augmented variable (*x*_5_) based on the ESO determines the system disturbance. According to Fig 5A, the observed disturbance (blue dashed line) tracks the actual disturbance (yellow continuous line) with a small error. The simulation results show that the RMS error is 2.290 mm, and the mean error is only 0.289 mm, much smaller than the excitation amplitude (50 mm). The change in sprung mass displacement with time is shown in Fig 5B. Looking at this figure more closely, one can see that the first state variable (*x*_1_) change follows the sinusoidal rule. The displacement of the vehicle body is the largest when the car is equipped with only passive suspension. The simulation results show that their peak and RMS values are 52.550 mm and 36.920 mm, respectively. Once the passive suspension system is replaced by the active suspension system controlled by the PID algorithm, these values decrease to 23.650 mm (peak value) and 15.930 mm (RMS value). A more significant improvement is seen when the ADRCBF algorithm is applied to control the vehicle suspension. According to the study’s findings, the peak value of the vehicle body displacement decreases sharply to 3.757 mm, while the RMS value reaches only 2.562 mm. The state variable values observed by ESO are equal to those obtained by the ADRCBF algorithm (results have been rounded).

Fig 5C explains the change in suspension travel. The suspension system needs to work harder to reduce the vibration of the sprung mass; that is, the suspension travel needs to change more. The simulation results show that suspension travel is the largest (equivalent to road disturbance) when the suspension system is controlled by the technique proposed in this article. The error between the actual signal (ADRCBF) and the observed signal (ESO) is only 1.162% (peak value) and 1.606% (RMS value). In contrast, the passive suspension travel is minimal, causing the vibration of the vehicle body to increase (Fig 5B). The suspension travel generally does not exceed its maximum limit (from 100 to 150 mm, depending on the specific vehicle).

**Fig 6:**
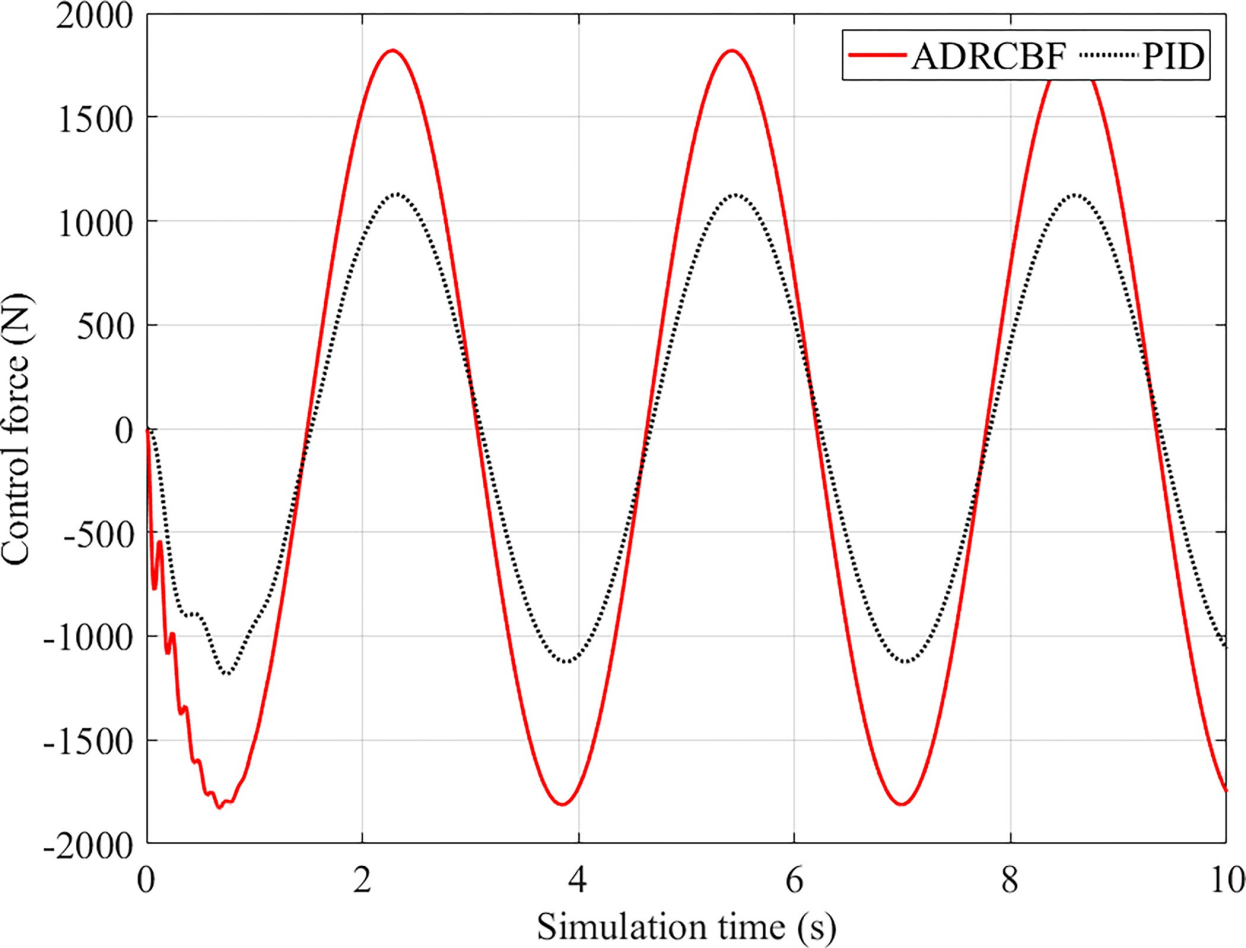
Control force (sinusoidal excitation).

The smoothness and comfort of the vehicle are evaluated by sprung mass acceleration. The simulation results illustrated by the lines in Fig 6D show that the vehicle body acceleration increases abruptly to a peak value in the first phase, up to 0.920 m/s^2^ (Passive) and 0.880 m/s^2^ (PID). This value decreases to 0.292 m/s^2^ once the conventional suspension is replaced by the active suspension controlled by the ADRCBF technique. Then, the sprung mass acceleration value gradually decreases and oscillates steadily around an equilibrium threshold. Compared to the passive suspension, the RMS acceleration obtained by the PID controller is 78.571%, while the value obtained by the ADRCBF is only 19.748%. In addition, the maximum error between the measured and observed signals is only 2.397%, while the RMS error is approximately zero. According to the ISO 2631 standard proposed by the International Standard Organization (ISO), ride comfort is evaluated through the value of the vehicle body acceleration. The calculation results show a good feeling when the proposed method controls the suspension.

The response of the control force is depicted in Fig 6. As a result, the value of the control force increases sharply when the ADRCBF technique controls the system. The peak value of the control force is up to 1828.320 N, which is 645.791 N higher than that of PID control. The RMS value of the control force obtained by the proposed controller is 1.625 times larger than that of a conventional PID controller. Therefore, the performance of the system is improved.

The simulation results obtained in this case are listed in Table 5.

**Table 5:** Calculation results (sinusoidal excitation).

	ADRCBF		ESO		PID		Passive	
	Max	RMS	Max	RMS	Max	RMS	Max	RMS
Sprung mass displacement (mm)	3.757	2.562	3.757	2.562	23.650	15.930	52.550	36.920
Suspension travel (mm)	46.550	32.680	46.009	32.155	27.937	19.545	6.234	1.952
Sprung mass acceleration (m/s^2^)	0.292	0.047	0.299	0.047	0.880	0.187	0.920	0.238
Control force (N)	1828.320	1294.054			1182.529	796.524		

### 3.2. Step excitation

**Fig 7:**
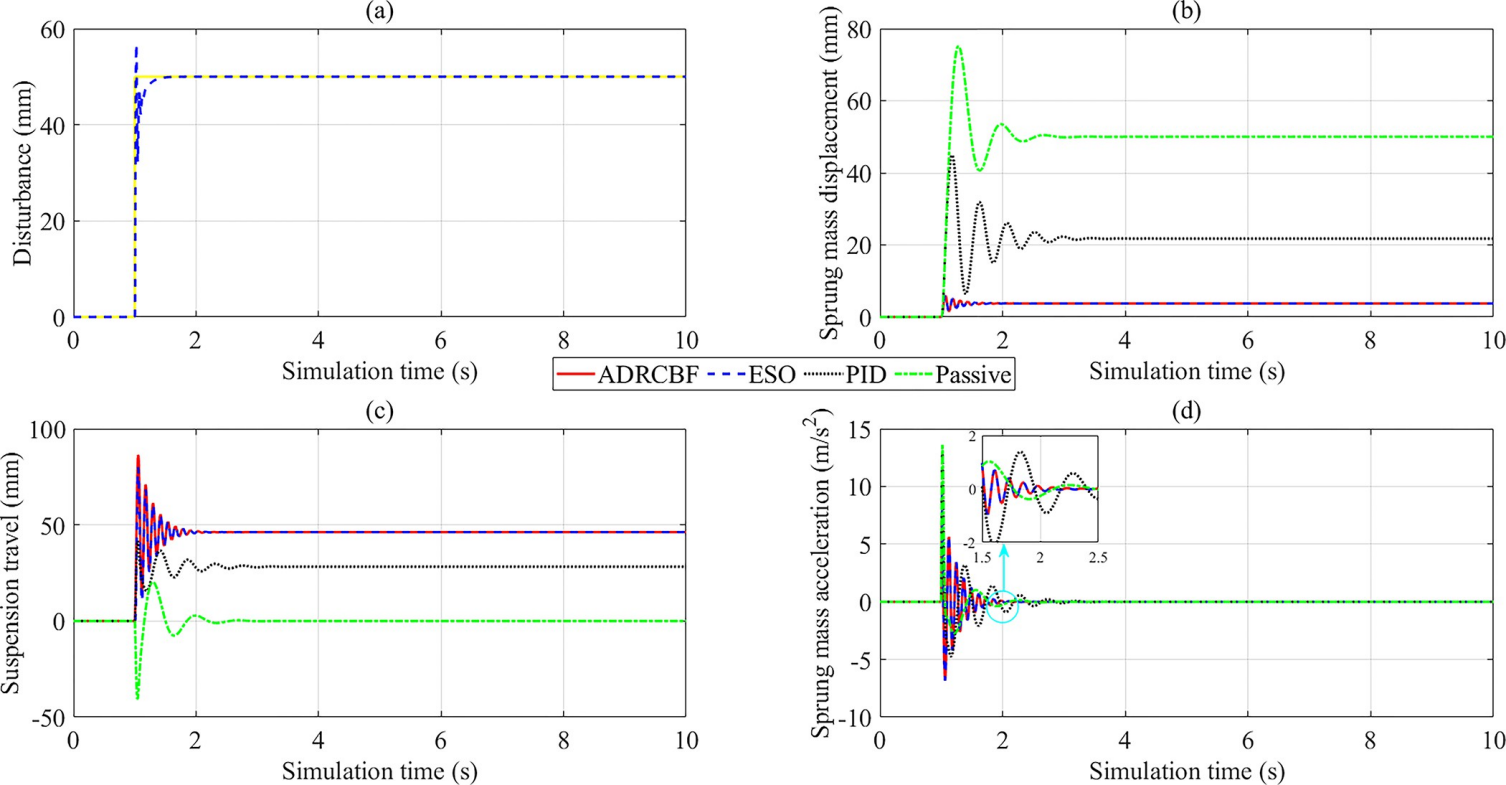
Simulation results (step excitation). (a) Observed disturbance. (b) Sprung mass displacement. (c) Suspension travel. (d) Sprung mass acceleration.

While sinusoidal excitation is used to investigate the system’s stability repeatedly over a certain period, step excitation is utilized to evaluate the overshoot phenomenon and the response speed of the control system. In this case, the observed disturbance follows the reference signal with a small error (Fig 7A). The overshoot phenomenon occurs slightly when the excitation signal reaches its peak; however, its influence is negligible. The change in vehicle body displacement is illustrated in Fig 7B. According to this description, the vehicle body displacement peaks at 75.201 mm, then decreases to 50 mm (Passive). Once the active suspension is used to replace the conventional mechanical suspension, the peak displacement value decreases to 44.933 mm, while the steady-state value is approximately 22 mm (PID). On the contrary, the vibration of the vehicle body is significantly improved when applying the proposed technique to control the suspension system, which reduces the sprung mass displacement to 5.964 mm. Compared with PID and Passive situations, the signal obtained from ADRCBF can converge earlier and quickly reach a steady state after only a tiny oscillation period.

According to Fig 7C, the suspension travel achieved by the proposed algorithm is superior to the other two situations (PID and Passive). This demonstrates the system’s advanced adaptability under external disturbances, which reduces the vibration of the car body (Fig 7B). The value of suspension travel is still within the allowable range. The last subplot depicts the acceleration of the car body. Looking more closely at Fig 7D, we can see that the acceleration value increases rapidly to the peak and gradually decreases to zero. The simulation results show that the car body’s acceleration can reach 13.604 m/s^2^ if it only uses the traditional passive suspension system. Even when the PID technique controls the suspension system, the peak value of the acceleration only decreases slightly to 13.496 m/s^2^. Compared to the two situations above, the performance of the suspension system is significantly improved once the ADRCBF technique is applied to control the system. According to the simulation findings, the peak value of the vehicle body acceleration is only 7.337 m/s^2^ and then rapidly decreases to zero. In this case, the observed signal follows the actual signal with a small error, except when the excitation pulse reaches its maximum. Although this error only occurs for a minimal time interval with a small value, it is considered a limitation of this study. The vibrations mentioned in this case are not continuous, so the RMS value is not considered.

**Fig 8:**
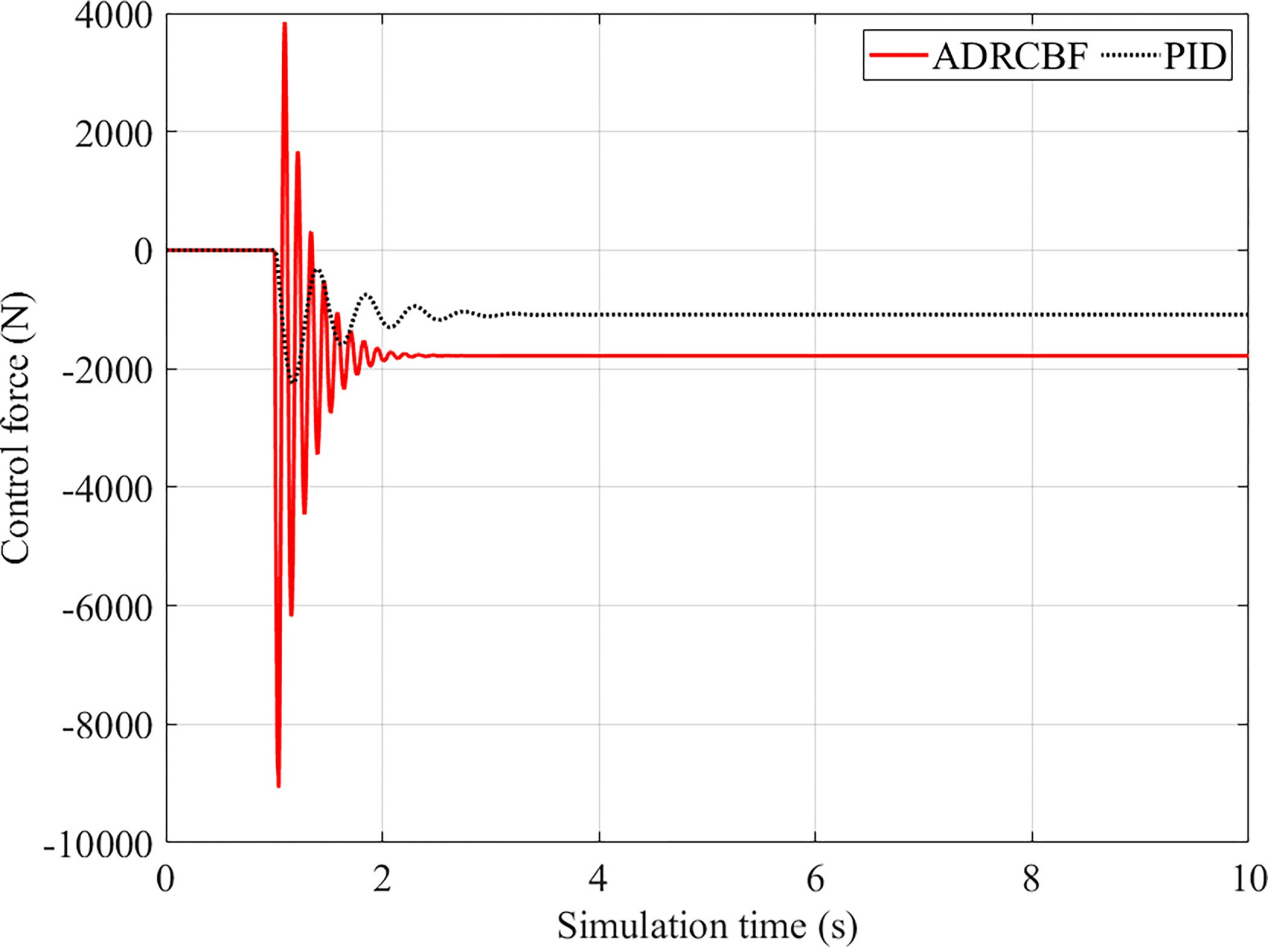
Control force (step excitation).

In this case, the control force changes abruptly under step excitation (Fig 8). Then, the value of the control force rapidly decreases to a steady state. The peak value of the control force is smaller than its limitation (about 15÷17 kN).

The results obtained from the simulation process are listed in Table 6.

**Table 6:** Calculation results (step excitation).

	ADRCBF		ESO		PID		Passive	
	Max	RMS	Max	RMS	Max	RMS	Max	RMS
Sprung mass displacement (mm)	5.964		5.955		44.933		75.201	
Suspension travel (mm)	86.344		79.908		41.699		40.678	
Sprung mass acceleration (m/s^2^)	7.337		10.108		13.496		13.604	
Control force (N)	9074.148				2246.645			

### 3.3 Random excitation

The above two types of road disturbances are often used in simulation problems. In fact, road disturbances are random, and their profile cannot be accurately determined. A random excitation generated by a white noise source is proposed in this case. The amplitude and frequency of the excitation signal are large, causing the vehicle to vibrate intensely.

**Fig 9:**
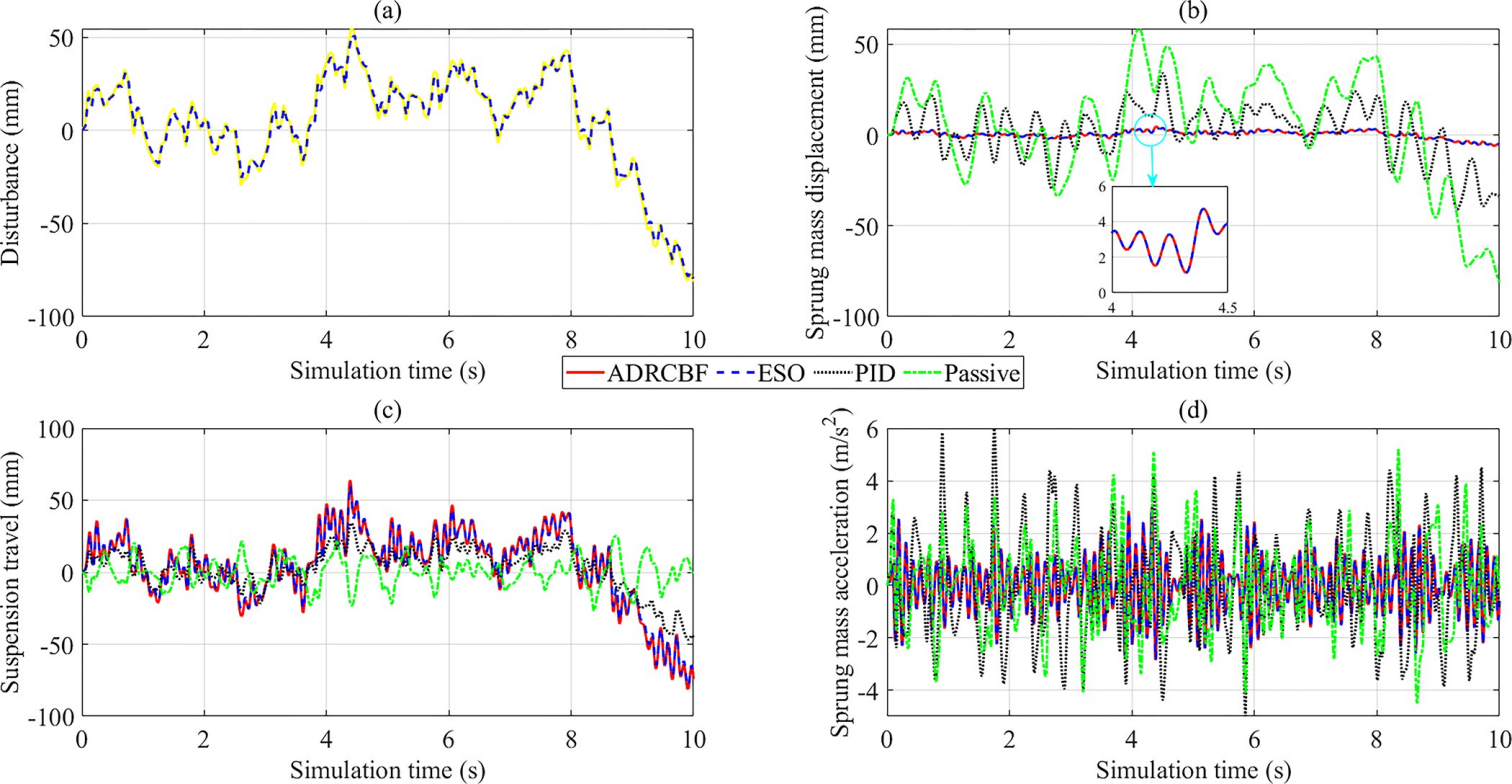
Simulation results (random excitation). (a) Observed disturbance. (b) Sprung mass displacement. (c) Suspension travel. (d) Sprung mass acceleration.

The simulation results shown in Fig 9A demonstrate that the ESO’s observed disturbance has high accuracy. Its RMS error and mean error are only 2.482 mm and 0.171 mm, respectively, much smaller than the excitation’s amplitude (up to more than 81 mm). Under the influence of a random disturbance, the vehicle body vibrates continuously with a large amplitude. According to the description in Fig 9B, the vehicle body vibration is the largest when the vehicle has only the passive suspension system. The peak and RMS values of the sprung mass displacement are 81.197 mm and 29.214 mm, respectively. These figures are reduced to 41.104 mm and 15.005 mm when the PID technique is applied to control the automotive suspension. A significant improvement is seen when the ADRCBF method is applied to the active suspension, resulting in a sharp reduction in the sprung mass displacement to 5.928 mm (peak value) and 1.978 mm (RMS value). The error between the signal observed by the ESO and the actual signal is only about 0.001 mm (results have been rounded).

Under the influence of a random disturbance, the suspension travel changes continuously over time. The suspension system needs to work harder to suppress the body vibration, causing the suspension travel to increase. According to Fig 9C, the most effective response belongs to the ADRCBF controller, which closely follows the road disturbance (Fig 9A). The RMS error between the observed and actual signals is only 3.374%. The peak value of suspension travel is only about 81 mm (ADRCBF), which is lower than the limited value. This proves that the suspension system is capable of stable operation under strong vibration conditions.

In this case, the vehicle body acceleration is large (Fig 9D). This value can reach 5.215 m/s^2^ (peak value) and 1.694 m/s^2^ (RMS value) if the car only has mechanical suspensions. These figures even increase to 6.031 m/s^2^ and 2.038 m/s^2^, respectively, if the mechanical suspension is replaced by the active suspension controlled by the PID technique. This shows that the performance of the PID controller is severely degraded when the system faces external solid disturbances. The ADRCBF algorithm provides superior performance in eliminating the effects of road disturbance, reducing the vehicle body acceleration to 2.977 m/s^2^ (maximum value) and 1.041 m/s^2^ (RMS value). In addition, the error estimated by the ESO is only about 2.150% compared to the actual value. The results of the calculations are compared with the ISO 2631 standard, and it is concluded that the vehicle vibrations are moderate when the ADRCBF technique controls the system. Passengers can tolerate this level for up to 24 consecutive hours. On the contrary, intense vibrations are seen when the suspension is controlled only by the traditional PID technique.

**Fig 10:**
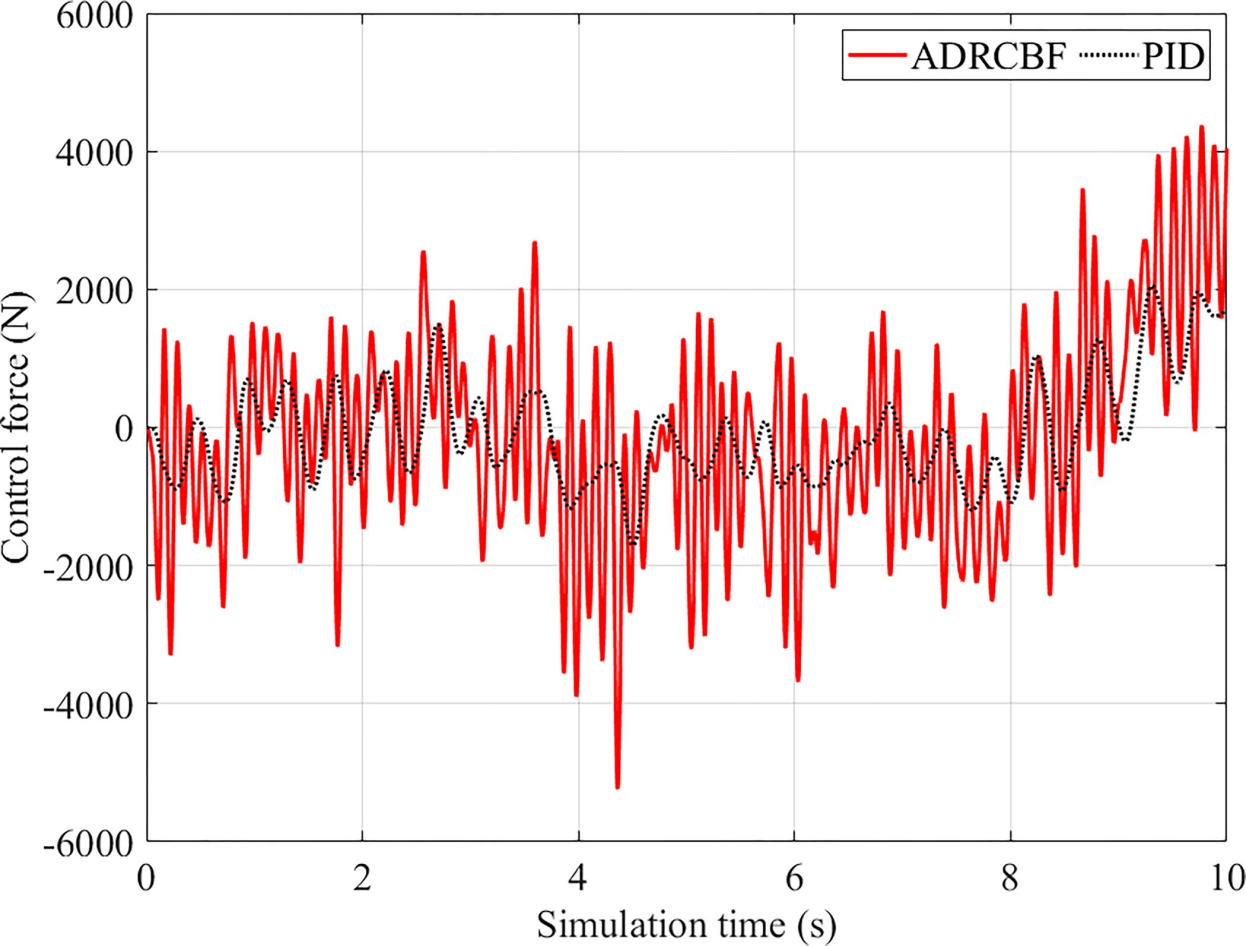
Control force (random excitation).

Fig 10 depicts the variation of control force under random excitation. In general, the response of the proposed controller is better than that of PID control. As a result, the range of control force is doubled when the ADRCBF technique controls the system. These results are listed in Table 7.

**Table 7:** Calculation results (random excitation).

	ADRCBF		ESO		PID		Passive	
	Max	RMS	Max	RMS	Max	RMS	Max	RMS
Sprung mass displacement (mm)	5.928	1.978	5.929	1.978	41.104	15.005	81.197	29.214
Suspension travel (mm)	81.165	26.054	78.856	25.175	46.958	15.258	26.977	10.128
Sprung mass acceleration (m/s^2^)	2.977	1.041	3.041	1.041	6.031	2.038	5.215	1.694
Control force (N)	5237.356	1491.440			2055.158	750.245		

Overall, the proposed algorithm outperforms the traditional PID regarding control performance. Table 7’s results demonstrate an improvement of 86.818% in the RMS value of sprung mass displacement and 48.921% in the RMS value of sprung mass acceleration. In addition, the value of vehicle body acceleration achieved by the proposed technique is reduced compared to PID-GA (introduced in [6]). Additionally, the vehicle body displacement is significantly reduced compared to the in-loop genetic algorithm discussed in [7]. Finally, the effects of chattering and sensor noise are wholly eliminated compared to [28–31].

Based on the results, some observations are made as follows:

+ To reduce vehicle vibrations, the suspension system must work hard, i.e., the suspension travel must follow road disturbances.

+ The displacement and acceleration of the vehicle body are significantly improved when the proposed method is applied to control the car’s active suspension system.

+ The PID controller only provides sufficient performance in one or some cases that are suitable for the selected parameters. On the other hand, the proposed algorithm’s practical response ability ensures the system’s adaptation in many investigated conditions, even when the road disturbance changes.

+ The observed signals have high accuracy in many different operating conditions.

+ The obtained computational results are highly feasible. However, the influence of nonlinearity and uncertainty has not been considered in this work. This could potentially lead to discrepancies in the experimental results.

+ In general, the structure of the controller proposed in this article is simple, while the stability and adaptability of the system are still guaranteed. In fact, automotive control systems are closely related (for instance, the braking system combined with the advanced stability system, the steering system combined with the anti-roll system, and others). As a result, simplifying the control algorithm opens up many opportunities for designing practical products.

## 4. Conclusion

In this article, we have introduced the control algorithm for the active suspension system, which is formed by combining the active disturbance rejection control technique and fuzzy computing. The vehicle’s vibration is illustrated by a dynamic model designed based on six state variables (five system variables and one augmented variable). The extended state observer is established to estimate the changes in system disturbances and other state variables instead of measuring them directly with sensors.

The performance of the proposed algorithm is verified by simulation and comparison with other algorithms. The numerical calculation results show that the acceleration and displacement of the vehicle body are significantly reduced when applying the ADRCBF technique, compared with PID and Passive. In addition, the signals observed by the ESO have high accuracy and acceptable errors.

Despite the high efficiency of the algorithm in controlling the system, some limitations still exist. Firstly, the overshoot phenomenon in the observed signal still exists under the influence of step disturbance, which increases the estimation error. Secondly, the actuator model has been linearized to simplify the calculation process, which generates an error between the simulation and actual results. Thirdly, the effects of high nonlinearity and uncertainty must be considered in specific investigation cases. Finally, the fuzzy controller parameters must be optimized to improve the system performance instead of being selected based on experience. In the following works, we plan to establish a fully dynamic model that considers the effects of nonlinearity and uncertainty to solve the second and third problems. The first and fourth problems can be resolved by applying optimization techniques to find the ideal parameter values for the controller. In addition, experiments are necessary to demonstrate the performance of the proposed algorithm.
